# Effects of mouth breathing on maxillofacial and airway development in children and adolescents with different cervical vertebral maturation stages: a cross-sectional study

**DOI:** 10.1186/s12903-022-02234-x

**Published:** 2022-05-23

**Authors:** Jiahua Li, Ziyi Zhao, Leilei Zheng, Baraa Daraqel, Jing Liu, Yun Hu

**Affiliations:** 1 Stomatological Hospital of Chongqing Medical University, No.426 Songshibei Road, Yubei District, Chongqing, 401147 China; 2 Chongqing Key Laboratory of Oral Diseases and Biomedical Sciences, Chongqing, China; 3 Chongqing Municipal Key Laboratory of Oral Biomedical Engineering of Higher Education, Chongqing, China; 4 Qingdao Stomatological Hospital, Shandong, China

**Keywords:** Cervical vertebral maturation, Maxillofacial development, Airway development, Mouth breathing

## Abstract

**Background:**

To examine the influence of mouth breathing on maxillofacial and airway development in children and adolescents with different cervical vertebral maturation stages.

**Methods:**

Lateral cephalometric radiograph of a total of 120 children and adolescents, 64 girls and 56 boys (7–15 years old), diagnosed with mouth breathing were examined. Maxillofacial hard tissue, soft tissue and airway measurements were obtained using both manual and digital techniques. Independent samples t-test was performed to compare the difference between the measured indexes and the standard values.

**Results:**

As for maxillofacial hard tissue, SNB (CS1–CS5), GoGn (CS1–CS5), ArGoNa (CS1–CS5), ArGo (CS1–CS2) and SNA (CS1–CS2) in mouth breathing children and adolescents were below the standard values (P < 0.05). NGoMe (CS1–CS5), SN-MP (CS1–CS4), SN-PP (CS1–CS4), PP-MP (CS1–CS3) and SN-GoGn (CS1–CS2) in mouth breathing children and adolescents were above the standard values (P < 0.05). As for maxillofacial soft tissue measurements, H angle (CS1–CS5), lower lip length (CS1–CS5), upper lip protrusion (CS1–CS5), upper lip length (CS1–CS4), lower lip protrusion (CS1–CS3), surface Angle (CS2–CS3) and nasolabial angle (CS2) in mouth breathing children and adolescents were above the standard values with statistically significance (P < 0.05). As for airway measurements, PAS (CS1, CS2, CS5) in mouth breathing children and adolescents was above the standard value with statistical significance (P < 0.05).

**Conclusions:**

Mouth breathing had a real effect on maxillofacial and airway development, which differed among mouth breathing children and adolescents with different cervical vertebral maturation.

**Supplementary Information:**

The online version contains supplementary material available at 10.1186/s12903-022-02234-x.

## Background

Chronic rhinitis, hypertrophy of the adenoid, palatine tonsils and deviated nasal septum are the frequent cause of upper respiratory obstruction, which forces children to breathe through their mouths [1]. Researches showed that mouth breathing incidence in children was 17–50% [2, 3]. Abnormal breathing patterns of mouth breathing leads to posteroinferior rotation of the mandible, inducing a prolonged imbalance of oropharynx muscle activity. Cranial and maxillofacial muscles produce a series of adaptive alterations, affecting the jaw's growth and development and leading to malocclusion [4, 5].

The craniofacial characteristics such as anterior overbite, deep overjet, poor lip seal, mandibular retrusion, and airway stenosis tend to worsen with the dentofacial growth of children with mouth breathing [6]. Stahl et al. investigated the relationship between cervical bone maturity and mandibular growth to infer that craniofacial growth in subjects with untreated Class II malocclusion had significantly smaller increases in mandibular length at the growth spurt and during the overall observation period [7]. Facial profile changes, the diagnosis of jaw bone disharmony, the most suitable intervention or treatment time, and the stability of the curative effect depend heavily on the growth characteristics and growth spurt of the maxilla and mandible. Therefore, understanding maxillofacial development and morphological characteristics in different stages is of great significance to the treatment planning, the control of tissue reconstruction, and the long-term prognosis. Luca [8] et al. took a cephalometric comparison about skeletal and dental features in ninety-eight children aged 7–12 years old who were split into two groups: mouth breathing secondary to nasal septum deviation and nasal breathing controls, finding that mouth breathing children displayed an increase of palatal height and overjet and upper and lower anterior facial height, a significantly retrognathic position of the maxilla and mandible, and the narrow of maxillary intermolar width. In addition, it was proposed that most mouth breathing children presented a class II skeletal malocclusion and cross-bite occurred more frequently than in the nasal breathing children. This is consistent with the traditional view that the facial type of mouth breathing children is mostly manifested as maxillary protrusion, mandibular retraction.

Helena [9], Isabel [10], Maria [11–13], Sara [14] et al., studied the influence of mouth breathing caused by upper respiratory tract obstruction, such as allergic rhinitis, nasal obstruction, adenoid/tonsil hypertrophy on maxillofacial development in children. Compared with the nasal breathing children, mouth breathing children showed the statistical insignificance of SN-PP and the increase of PP-MP, SN-MP, SN-OP, which reflected the minor secondary effect on maxilla, the occlusal plane steepened and the posteroinferior rotation of the mandible. The values of SPAS, PAS and other airway indicators in the mouth breathing group were decreased, indicating that airway stenosis was caused by mouth breathing in the process of posterior rotation of mandible.

The influence of mouth breathing on the maxillofacial development is still unsettled. The research results on the effects of mouth breathing on the anterior lower height of the face and the position of the maxilla [10, 15–17]. Contrary to previous studies [1, 18], Bernardo [19] et al. proposed that the ANB Angle of mouth breathing children was significantly larger than that of nasal breathing controls in both primary dentition and mixed dentition, while the SNB Angle decreased. Doron [20] et al. proposed that retrusive mandible in mouth breathing children was mainly manifested by deepened overjet and SNB Angle, which was inconsistent with Bernardo's results.

Compared with chronological age, skeletal age can more accurately reflect individual growth and maturity [21–23]. Maturational stages refer to specific developmental events identified on hand-wrist or cervical x-rays that relate directly to the progression of maturation during childhood and adolescence. Each progressive stage represents an increasing percentage of total facial skeletal growth completed. Although cervical vertebrae x-rays do not allow for such definitive evaluation as hand-wrist x-rays, the cervical vertebra maturity assessment system has a unique advantage that cephalometric radiographs are routine for orthodontic analysis and treatment planning avoiding additional X-ray exposure [24]. Hassel and Farman [25], Garcia-Fernandez et al. [26] have reported that cervical vertebral maturation analysis was comparable to hand-wrist analysis for assessing skeletal maturity, and it had high reliability and validity. And the variation of the cervical vertebra ossification center is more obvious to observe in the development period due to its fewer amount [27].

Due to the ongoing controversy and the lack of cervical vertebral maturation method in the effects of mouth breathing on maxillofacial and airway development, in this cross-sectional study, we made the statistical comparisons for cephalometric measurements to explore the craniofacial and airway growth changes in children and adolescents with mouth breathing, as defined by the cervical vertebral maturation method. Our study has three null hypotheses. The first null hypothesis is’mouth breathing affects the maxillofacial hard tissue development throughout all the growth and development period (for the cervical vertebrae maturation [CVM] method)’. The second null hypothesis is ‘mouth breathing affects the maxillofacial soft tissue development throughout all the growth and development period (for the cervical vertebrae maturation [CVM] method)’. The third null hypothesis is’mouth breathing affects the airway development throughout all the growth and development period (for the cervical vertebrae maturation [CVM] method)’.

## Methods

### Study design and setting

This cross-sectional study was conducted with the approval of the ethical committee of the Stomatological Hospital of Chongqing Medical University (ID: CQHS-REC-2020(LSNO.49)) in compliance with the Helsinki Deceleration and the Strengthening the Reporting of Observational Studies in Epidemiology (STROBE) guidelines (Additional file 1). All the information was collected with informed consent from the guardians of the children and adolescents.

### Study participants

According to the sample content calculation formula N = K*Q/P(K = 100,Q = 1-P when the allowed error is 20%,P is the expected incidence rate, and the incidence rate of adenoid hypertrophy is 49.7%), a total of 120 mouth breathing children and adolescents were retrospectively examined and included in this study during the period from December 2018 to September 2019. The age limit for participating in the study was 7–15 years of age. Combined with the results of the guardian's inquiry and clinical examination, the patient who fulfill the following two conditions could be classified as a mouth breath child or adolescent [10]: they had the habit of breathing through their mouth at rest or during sleep. At the same time, the child or adolescent was in a resting position with a double-sided mirror placed under the nostril observed for the presence of fogging or water vapor in the mouth side, or with a cotton wool placed under the nostril observed fluttering. Children and adolescents with the nasal inflammatory lesions or space-occupying lesions, history of other persistent oral habits, history of adenoidectomy or tonsillectomy, maxillofacial surgery history or history of orthodontic treatment were excluded from the analysis.

### Variables and data measurement

**Fig. 1:**
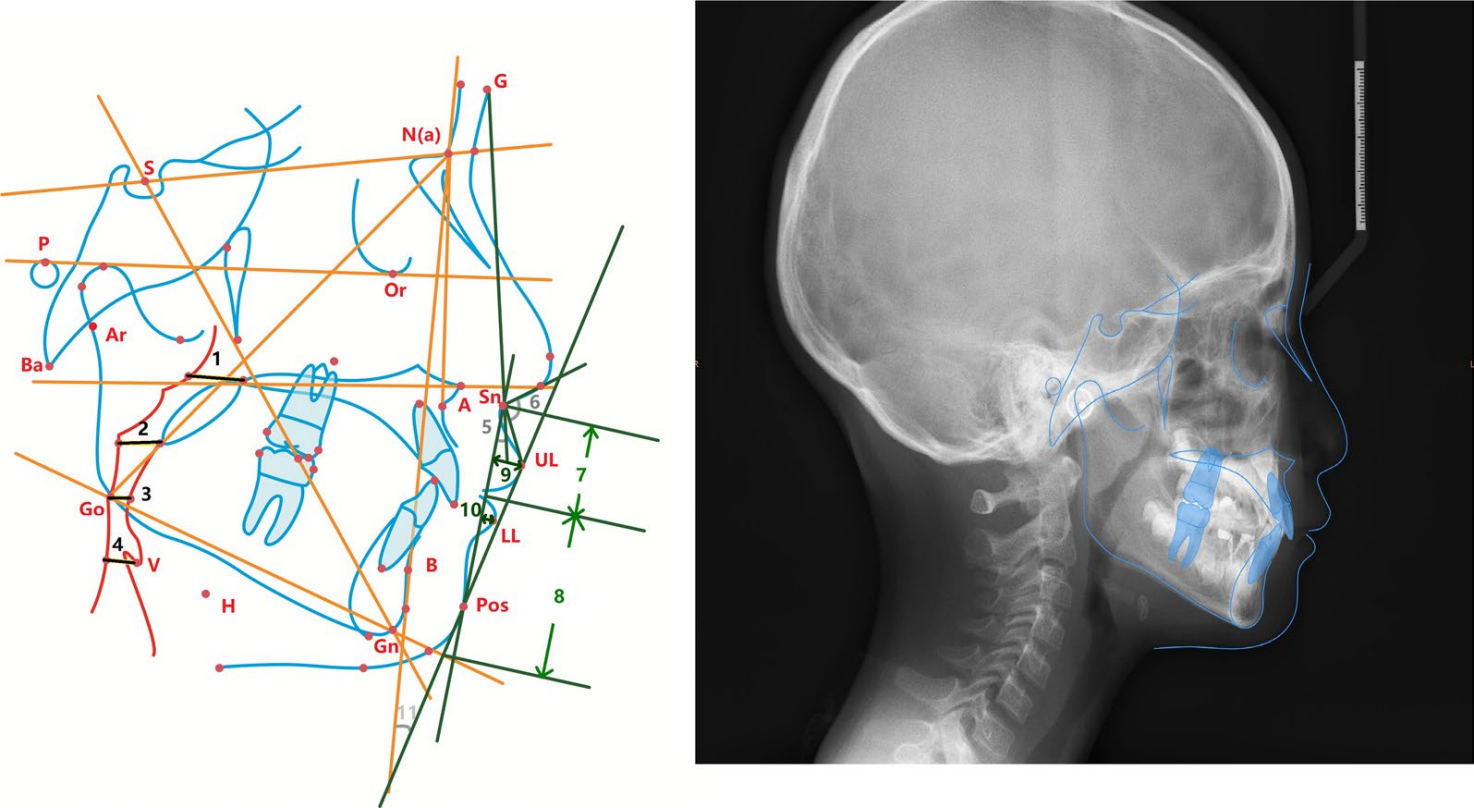
Cephalometric reference planes. Cephalometric index. 1 = PNS-UPW; 2 = U-MPW; 3 = PAS; 4 = V-LPW; 5 = surface Angle; 6 = nasolabial Angle; 7 = length of upper lip; 8 = length of lower lip; 9 = upper lip protrusion;10 = lower lip protrusion;11 = H Angle

All patients in the current study were subjected to X-ray imaging in the intercuspal position. Cephalograms were routinely obtained with an X-ray diagnosis system (Kodak 9000; Kodak, Rochester, NY, USA) at a voltage of 62 kV, current of 8 mA and distance of median sagittal plane to the X-ray source of 154.5 cm. The digitized X-ray cephalograms were uploaded into the Dolphin 3D software (version 11.0; Dolphin Imaging, Chatsworth, CA). The detailed craniofacial hard tissue landmarks and measurement items that were established are shown in Fig. 1 and Table 1. 120 Subjects were then divided into six stages CS1-CS6 according to the method of cervical vertebral maturation assessment was described in Reilly’s study [28]. All Cephalometric measurements were measured twice in three months interval by two trained examiners using Dolphin 3D software, and the intra-class correlation coefficient was applied to analyze the internal reliability of observers. The mean of the two measured values was used for the final statistical analysis.

**Table 1:** Indicator definitions and standard values were included

Item	Definition	Diagnostic value	Normal value
Craniofacial measurements			
SNA	Angle formed by the sella-nasion line and line N-point A	Anteroposterior position of the maxilla in relation to the skull base	82°
SNB	Angle formed by the sella-nasion line and line N-point B	Anteroposterior position of the mandible in relation to the skull base	80.9°
ANB	Differences between the SNA and SNB angles	Relation between maxilla and mandible	3°
Y	Anteroinferior angle between Y axis and FH plane	Protrusion of mental region, as well as the growth direction	67°
FH-MP	Angle formed by the intersection of the FH plane and MP plane	Mandibular inclination	25.9°
SN-MP	Angle formed by the intersection of the SN plane and MP plane	Mandibular inclination	33°
PP-MP	Angle formed by the intersection of the PP plane and MP plane	Mandibular inclination	25°
SN-PP	Angle formed by the sella-nasion line and palatal plane	The degree of the maxilla inclination in relation to the anterior cranial base	8°
SN-GoGn	Angle formed by the sella-nasion line and mandibular plane	Inclination of the mandibular plane in relation to the skull base	33°
GoGn	Linear distance between the gnathion and gonion	The length of mandibular body	75.4°
ArGo	Linear distance between the articulare and gonion	The length of mandibular ramus	39.3°
ArGoNa	Angle formed by the articulare, gonion and nasion	The inclination of mandibular ramus	58.1°
NaGoMe	Angle formed by the nasion, gonion and menton	The inclination of mandibular body	69.7°
NSAr	Angle formed by the nasion, sella and articulare	Extent of inclination of mandibular body	124°
SArGo	Angle formed by the sella, articulare and gonion	Extent of anterior inclination of the mandible	144.9°
Airway measurements			
PNS-UPW	Distance between PNS and UPW	Nasopharyngeal airway space	21.58 mm(M) 23.06 mm(W)
U-MPW	Distance between U and MPW	Upper oropharyngeal airway space	9.92 mm(M) 10.22 mm(W)
V-LPW	Distance between V and LPW	Laryngeal airway space	13.97 mm(M) 15.01 mm(W)
PAS	Width of the airway space along the Go-B line	Pharyngeal Airway Space	10 mm
Soft tissue measurements			
Surface angle	Angle formed by the G, Sn and Pos	Extent of protrusion of soft tissue	12°
Nasolabial angle	Angle formed by the Prn, Sn and Ls	Relationship between the upper lip and columella Nasi	102°
Upper lip length	Linear distance between perpendiculars drawn to SN-POS from Sn and Stms respectively	Length of upper lip	21.5 mm
Lower lip length	Linear distance between perpendiculars drawn to SN-POS from Mes and Stmi respectively	Length of lower lip	47 mm
Upper lip protrusion	Linear distance between UL and Sn-Pos	Extent of protrusion of upper lip	3 mm
Lower lip protrusion	Linear distance between LL and Sn-Pos	Extent of protrusion of lower lip	4.2 mm
H angle	Angle formed by the Pos-UL and NB	Relationship between the soft tissue chin and lip	10°

### Statistical analysis

Independent samples t-test was conducted to compare the difference between the measured index and the standard value, using SPSS software (version 22.0) for statistical analysis of measurements consistent with normality and homogeneity of variance. The significance level was set at 0.05.

## Results

According to the cervical vertebral maturation assessment, there were 45 CS1 cases, 33 CS2 cases, 21 CS3 cases, 9 CS4 cases, 12 CS5 cases, and 0 case of CS6.

### Maxillofacial hard tissue measurements

As shown in Table 2, SNB, GoGn and ArGoNa from CS1 through CS5 were below the standard values and had statistical significance. The measured values of ArGo and SNA were below the standard values and were statistically significant, only in CS1 and CS2 stages. The measured values of NGoMe (interval CS1–CS5), SN-MP (interval CS1–CS4), SN-PP (interval CS1–CS4), PP-MP (interval CS1–CS3) and SN-GoGn (interval CS1–CS2) were above the standard values and statistically significant. There was no statistical significance in other hard tissue measurements indexes of the maxillofacial region.

**Table 2:** Measurements of hard tissue indexes at different cervical vertebral maturation stage

Item	CS1		CS2		CS3		CS4		CS5	
	¯X ± S	P value	¯X ± S	P value	¯X ± S	P value	¯X ± S	P value	¯X ± S	P value
SNA	79.75 ± 3.42	0.000	80.73 ± 2.36	0.004	81.60 ± 2.76	0.512	80.13 ± 3.67	0.165	81..00 ± 2.64	0.215
SNB	74.69 ± 5.05	0.000	74.59 ± 4.70	0.000	75.82 ± 5.51	0000	76.40 ± 3.18	0.003	78.21 ± 3.78	0.031
ANB	4.53 ± 2.27	0.000	5.36 ± 1.97	0.000	5.72 ± 5.20	0.027	3.73 ± 2.91	0.473	2.81 ± 1.72	0.715
Y	71.83 ± 4.79	0.000	72.22 ± 2.91	0.000	70.28 ± 5.45	0.012	72.56 ± 3.14	0.001	70.29 ± 4.07	0.017
SN-MP	39.13 ± 4.79	0.000	39.16 ± 4.36	0.000	36.94 ± 3.46	0.000	37.63 ± 3.85	0.007	35.43 ± 7.49	0.286
FH-MP	29.69 ± 4.21	0.000	29.82 ± 3.85	0.000	29.21 ± 3.88	0.001	28.33 ± 3,78	0.090	27.64 ± 6.76	0.393
SN-Ar	123.80 ± 5.23	0.796	124.42 ± 5.16	0.647	120.93 ± 9.10	0.137	123.52 ± 6.26	0.825	118.47 ± 11.47	0.123
SArGo	151.01 ± 5.67	0.000	150.44 ± 6.12	0.000	152.65 ± 5.27	0.000	153.00 ± 4.78	0.001	152.86 ± 4.04	0.000
ArGo	34.57 ± 3.41	0.000	35.29 ± 3.53	0.000	37.72 ± 3.97	0.083	38.61 ± 4.62	0.665	39.75 ± 4.62	0.742
PP-MP	28.72 ± 4.83	0.000	29.84 ± 4.53	0.000	27.91 ± 4.16	0.004	27.20 ± 4.34	0.167	25.27 ± 6.48	0.887
SN-PP	10.48 ± 2.69	0.000	9.32 ± 3.08	0.019	8.95 ± 1.89	0.032	9.44 ± 0.68	0.000	10.13 ± 3.53	0.060
SN-GoGn	36.4215 ± 4.75	0.000	36.43 ± 4.13	0.000	34.23 ± 3.47	0.119	34.96 ± 3.85	0.165	32.85 ± 7.31	0.944
ArGoNa	47.68 ± 3.99	0.000	48.18 ± 3.95	0.000	46.37 ± 3.87	0.000	45.62 ± 3.77	0.000	45.85 ± 3.82	0.000
NaGoMe	76.64 ± 4.62	0.000	76.07 ± 4.79	0.000	75.12 ± 4.65	0.000	75.21 ± 4.84	0.009	75.43 ± 6.19	0.008
GoGn	65.41 ± 5.60	0.000	65.65 ± 7.22	0.000	69.49 ± 6.90	0.001	67.34 ± 9.91	0.041	71.33 ± 4.5	0.010

### Maxillofacial soft tissue measurements

As shown in Table 3, among soft tissue measurement indexes, H angle, lower lip length and upper lip protrusion were above the standard values with statistically significance from CS1 through CS5. The upper lip length (interval CS1–CS4), the lower lip protrusion (interval CS1–CS3), and surface Angle (interval CS2–CS3) were above the standard values with statistical significance. The nasolabial angle was above the standard value with statistical significance in the CS2 stage.

**Table 3:** Measurements of soft tissue indexes at different cervical vertebral maturation stage

Item	CS1		CS2		CS3		CS4		CS5	
	¯X ± S	P value	¯X ± S	P value	¯X ± S	P value	¯X ± S	P value	¯X ± S	P value
Surface angle	14.72 ± 9.79	0.069	16.5274 ± 6.92	0.001	15.88 ± 6.35	0.011	15.82 ± 7.38	0.159	11.07 ± 4.74	0.511
Nasolabial angle	103.2193 ± 8.25	0.327	97.70 ± 11.12	0.034	102.44 ± 10.65	0.852	96.73 ± 15.43	0.335	101.30 ± 8.17	0.773
Upper lip protrusion	7.02 ± 1.85	0.000	9.62 ± 8.39	0.000	8.76 ± 6.51	0.001	11.23 ± 10.27	0.043	6.31 ± 1.62	0.000
Lower lip protrusion	5.72 ± 2.50	0.000	6.31 ± 1.84	0.000	6.27 ± 2.25	0.000	6.29 ± 3.34	0.097	5.27 ± 2.62	0.185
Upper lip length	18.73 ± 1.75	0.000	19.31 ± 1.74	0.000	19.82 ± 1.92	0.001	20.49 ± 2.54	0.268	19.6.9 ± 2.14	0.014
Lower lip length	38.47 ± 3.90	0.000	39.23 ± 4.10	0.000	40.84 ± 4.93	0.000	40.89 ± 4.77	0.005	4208 ± 4.66	0.004
H angle	20.30 ± 4.24	0.000	21.48 ± 3.36	0.000	21.28 ± 2.90	0.000	20.61 ± 5.50	0.000	17.93 ± 3.71	0.000
PAS	11.95 ± 3.77	0.001	12.26 ± 3.21	0.000	11.27 ± 3.82	0.144	11.04 ± 3.18	0.354	11.96 ± 2.69	0.028

### Airway measurements

As shown in Table 4, PAS was above the standard value with statistical significance in CS1, CS2 and CS5. Regarding gender, the male PNS-UPW was below the standard value with statistically significance from CS1 to CS3, and the male U-MPW was above the standard value with statistically significance only in CS2. The male V-LPW was not statistically significant in the whole stage. The female PNS-UPW was below the standard value with statistical significance in the whole stage of CS1-CS5, and the female U-MPW and V-LPW were below the standard values with statistically significant only in the CS1 stage.

**Table 4:** Measurements of airway indexes at different cervical vertebral maturation stage

Item	CS1				CS2				CS3				CS4				CS5			
	male	P	female	P	male	P	female	P	male	P	female	P	male	P	female	P	male	P	female	P
PNS-UPW	15.28 ± 4.53	0.000	14.77 ± 3.0	0.000	17.32 ± 5.76	0.006	16.19 ± 4.13	0.000	16.42 ± 4.61	0.010	16.34 ± 4.97	0.001	17.77 ± 1.70	0.061	12.92 ± 3.47	0.001	19.07 ± 5.70	0.330	15.57 ± 3.69	0.004
U-MPW	10.58 ± 2.99	0.339	9.87 ± 2.94	0.048	10.75 ± 1.71	0.001	9.88 ± 2.51	0.609	9.88 ± 2.77	0.965	9.13 ± 2.10	0.099	10.63 ± 2.47	0.667	9.92 ± 1.07	0.518	10.32 ± 2.82	0.745	10.23 ± 1.87	0.987
V-LPW	12.98 ± 3.79	0.258	13.10 ± 3.37	0.009	13.69 ± 3.58	0.743	13.51 ± 3.54	0.122	12.87 ± 3.65	0.392	12.72 ± 4.20	0.085	12.93 ± 5.41	0.772	12.93 ± 3.00	0.151	16.12 ± 3.37	0.180	15.07 ± 3.65	0.971

## Discussion

According to the review, studies on the effects of mouth breathing on craniofacial morphology and airway development in children were mainly based on cross-sectional data. They took mouth breathing children in a certain age stage or multiple age stages as the research objects [29]. Previous review articles suggested the correlation between dental development and skeletal maturation was strong, and CVM was a reliable method in predicting the pubertal growth spurt [30, 31]. There is a lack of study about craniofacial morphology and airway development in children and adolescents with mouth breathing based on the cervical vertebral maturation method.

In this study, included mouth breathing children and adolescents were classified into six stages (CS1–CS6) according to the cervical vertebral maturation method. The cephalometric analysis found that the maxillary protraction of mouth breathing children and adolescents was during CS1 and CS2, and the mandibular deficiency was throughout the whole stage from CS1 to CS5, reflected in the underdevelopment of the mandible body and the inferior-posterior rotation of the mandible. The inferior-posterior rotation of the maxilla was also observed in the stage from CS1 through CS4. Our first hypothesis has been accepted. The maxillary growth peak began at the quantitative cervical maturity stage 1 (QCVM Stage I), but then slowed down, while the maximum growth of mandible began at quantitative cervical maturity stage 2(QCVM Stage II), followed by quantitative cervical maturity stage 1(QCVM Stage I), suggesting that the peak of maxilla was earlier than that of mandible, and the growth period of mandible was longer than that of maxilla [32, 33]. Bisham proposed that the mandibular angle of adults shrunk substantially during the growth period, with a superior-anterior rotation [34]. These conclusions were consistent with our study results, indicating that mouth breathing had an important and varying impact on the maxilla and mandible development.

Regarding facial soft tissue development, the increasing trend of surface Angle in mouth breathing children and adolescents existed in CS2 and CS3 stages. The decrease of nasolabial angle only occurred during CS2. The increase of upper lip protrusion and the decrease of upper and lower lip length basically run through the whole period of children and adolescents' growth (CS1–CS5). Our second hypothesis has been accepted. This can be explained by the fact that the equilibrium effect between the lips and the teeth is lost as mouth breathing brings out the proclination of the upper anterior incisors and the parting of lips [35]. It was different from the previous conclusion that in the process of growth and development, the skeletal profile protrusion gradually decreased, and the soft tissue profile basically remained constant, verifying that the development of soft tissue had little correlation with the underlying hard tissue [36].

Regarding airway development, the nasopharyngeal airway in female mouth breathing children and adolescents was significantly narrowed throughout the whole period of CS1–CS5. The nasopharyngeal and upper oropharyngeal airway narrowing in male mouth breathing children and adolescents, and the upper oropharyngeal airway narrowing in female mouth breathing children and adolescents only existed in the developmental stage before CS3. The third hypothesis has been accepted. There was no statistical significance in the change of laryngopharynx airway in male mouth breathing children and adolescents. Female mouth breathing children and adolescents laryngopharynx airway was below standard value, only in CS1 period.

In conclusion, the effect of mouth breathing on the maxilla and mandibular ramus in children and adolescents mainly exists in the early growth and development. The shortening of mandibular body length and posteroinferior mandibular rotation were observed during the whole development of mouth breathing children and adolescents. The effect of mouth breathing on the upper lip protrusion, the shortening of upper and lower lip length and the retrogenia existed in each growth and development stage. Female nasopharyngeal stenosis was more likely to be affected than male at all stages of growth and development.

It is significant to stress the caution that should be taken while interpreting the results presented in this study, for the lack of control group and its limitations as a cross-sectional study regarding growth analysis, which is lack of sensitivity to individual variability. And the results may be varied by region on account of genetics and nutrition. Thus, it is suggested that longitudinal studies are performed in different populations, investigating the changes in hard tissue, soft tissue, and airway measurements between cervical vertebral maturation stages of mouth breathing children and adolescents.

## Conclusions

The effect of mouth breathing on maxilla and mandibular ramus mainly existed in the early growth and development stage, while the effect on lip could exist through the whole growth and development stage. With regard to nasopharyngeal airway narrowing, females were more susceptible to oral-respiratory effects than males.

## Supplementary Information

**Additional file 1.** STROBE Checklist.

## Data Availability

All data generated or analyzed during this study are included in the article.

## Abbreviations

CVM: Cervical vertebrae maturation
CS: Cervical stage
