# Supplementary material for: Effects of mouth breathing on maxillofacial and airway development in children and adolescents with different cervical vertebral maturation stages: a cross-sectional study
Source: BMC Oral Health. 2022 May 23;22:197. doi: 10.1186/s12903-022-02234-x (PMC9125826; doi:10.1186/s12903-022-02234-x)
Supplement: Supplementary file 1 — Additional file 1. STROBE Checklist. [file 12903_2022_2234_MOESM1_ESM.docx]

STROBE Statement—checklist of items that should be included in reports of observational studies

|  | Item No. | Recommendation | Page  No. | Relevant text from manuscript |
| --- | --- | --- | --- | --- |
| **Title and abstract** | 1 | (*a*) Indicate the study’s design with a commonly used term in the title or the abstract | 1 | Background: To evaluate the correlation between cervical vertebral maturation and oral, maxillofacial and airway development in mouth breathing children and adolescents.  Methods: Lateral cephalometric radiograph of a total of 120 children and adolescents (7-15 years old) diagnosed with mouth breathing were examined. Maxillofacial hard tissue, soft tissue and airway measurements were obtained using both manual and digital techniques. Independent samples t-tests were performed to compare the difference between the measured indexes and the standard values.  Results: Maxillofacial and airway development showed significant differences in both angular and linear measurements compared with the normal values (P<0.05), indicating that mouth breathing had a real effect on maxillofacial and airway development, which differed among mouth breathing children and adolescents with different cervical vertebral maturation.  Conclusions: The effect of mouth breathing on maxilla and mandibular ramus mainly existed in the early growth and development stage, while the effect on lip could exist through the whole growth and development stage. With regard to nasopharyngeal airway narrowing, females were more susceptible to oral-respiratory effects than males.  Keywords: cervical vertebral maturation; maxillofacial development; airway development; mouth breathing |
|  |  | (*b*) Provide in the abstract an informative and balanced summary of what was done and what was found |  |  |
| Introduction | | | |  |
| Background/rationale | 2 | Explain the scientific background and rationale for the investigation being reported | 1-2 | The craniofacial characteristics such as anterior overbite, deep overjet, poor lip seal, mandibular retrusion, and airway stenosis tend to worsen with the dentofacial growth of children with mouth breathing[6]. Stahl et al. investigated the relationship between cervical bone maturity and mandibular growth to infer that craniofacial growth in subjects with untreated Class II malocclusion had significantly smaller increases in mandibular length at the growth spurt and during the overall observation period[7]. Facial profile changes, the diagnosis of jaw bone disharmony, the most suitable intervention or treatment time, and the stability of the curative effect depend heavily on the growth characteristics and growth spurt of the maxilla and mandible. Therefore, understanding maxillofacial development and morphological characteristics in different stages is of great significance to the treatment planning, the control of tissue reconstruction, and the long term prognosis. Take the Angle classⅡmalocclusion which is common in the mouth breathing patients as an example. The timing of functional correction for patients with Angle classⅡmalocclusion has been a long-standing issue in orthodontic practice. Petrovic et al. showed that activators, Frankel appliances and that sort of functional appliances were most beneficial in prepuberty[8]. Others supported that the most suitable timing of functional appliances was pubertal growth spurt[9]. Thus, seeking the maxillofacial and airway development characteristics in mouth breathing patients could help orthodontists elevate their individualized diagnosis and treatment planning level to select the proper treatment methodologies and timing rationally. |
| Objectives | 3 | State specific objectives, including any prespecified hypotheses | 2 | Our study has three null hypotheses. The first null hypothesis is ’mouth breathing affects the maxillofacial hard tissue development throughout all the growth and development period (for the cervical vertebrae maturation [CVM] method)’. The second null hypothesis is ‘mouth breathing affects the maxillofacial soft tissue development throughout all the growth and development period (for the cervical vertebrae maturation [CVM] method)’. The third null hypothesis is ’mouth breathing affects the airway development throughout all the growth and development period (for the cervical vertebrae maturation [CVM] method)’. |
| Methods | | | |  |
| Study design | 4 | Present key elements of study design early in the paper | 2 | This cross-sectional study was conducted with the approval of the ethical committee of the Stomatological Hospital of Chongqing Medical University. |
| Setting | 5 | Describe the setting, locations, and relevant dates, including periods of recruitment, exposure, follow-up, and data collection | 2 | This cross-sectional study was conducted with the approval of the ethical committee of the Stomatological Hospital of Chongqing Medical University. |
| Participants | 6 | (*a*) *Cohort study*—Give the eligibility criteria, and the sources and methods of selection of participants. Describe methods of follow-up  *Case-control study*—Give the eligibility criteria, and the sources and methods of case ascertainment and control selection. Give the rationale for the choice of cases and controls  *Cross-sectional study*—Give the eligibility criteria, and the sources and methods of selection of participants | 3 | The inclusion criteria are as follows: (1)7-15 age range; (2) Oral respiratory history≧2 years; (3) No family history of maxillofacial deformity; (4) No bad habits such as tongue spitting; (5) Lateral cephalograms of good quality; (6) Children and adolescents diagnosed as mouth breather. The exclusion criteria were children and adolescents with: (1) Nasal inflammatory lesions or space-occupying lesions; (2) History of adenoidectomy or tonsillectomy; (3) Maxillofacial surgery history; (4) History of orthodontic treatment. |
|  |  | (*b*) *Cohort study*—For matched studies, give matching criteria and number of exposed and unexposed  *Case-control study*—For matched studies, give matching criteria and the number of controls per case |  |  |
| Variables | 7 | Clearly define all outcomes, exposures, predictors, potential confounders, and effect modifiers. Give diagnostic criteria, if applicable | 3 | All patients in the current study were subjected to X-ray imaging in the intercuspal position. Cephalograms were routinely obtained with an X-ray diagnosis system (Kodak 9000; Kodak, Rochester, NY, USA) at a voltage of 62 kV, current of 8 mA and distance of median sagittal plane to the X-ray source of 154.5 cm. The digitized X-ray cephalograms were uploaded into the Dolphin 3D software (version 11.0; Dolphin Imaging, Chatsworth, CA). The detailed craniofacial hard tissue landmarks and measurement items that were established are shown in Fig. 1 and Table 1. 120 Subjects were then divided into six stages CS1-CS6 according to the method of cervical vertebral maturation assessment was described in Reilly’s study |
| Data sources/ measurement | 8* | For each variable of interest, give sources of data and details of methods of assessment (measurement). Describe comparability of assessment methods if there is more than one group | *3* | *All patients in the current study were subjected to X-ray imaging in the intercuspal position. Cephalograms were routinely obtained with an X-ray diagnosis system (Kodak 9000; Kodak, Rochester, NY, USA) at a voltage of 62 kV, current of 8 mA and distance of median sagittal plane to the X-ray source of 154.5 cm. The digitized X-ray cephalograms were uploaded into the Dolphin 3D software (version 11.0; Dolphin Imaging, Chatsworth, CA). The detailed craniofacial hard tissue landmarks and measurement items that were established are shown in Fig. 1 and Table 1. 120 Subjects were then divided into six stages CS1-CS6 according to the method of cervical vertebral maturation assessment was described in Reilly’s study.* |
| Bias | 9 | Describe any efforts to address potential sources of bias | 3 | All Cephalometric measurements were measured twice in three months interval by two trained examiners using Dolphin 3D software, and the intra-class correlation coefficient was applied to analyse the internal reliability of observers. The average of the two measurements was used for the final statistical analysis. |
| Study size | 10 | Explain how the study size was arrived at | 3 | According to the sample content calculation formula N=K*Q/P(K=100,Q=1-P when the allowed error is 20%,P is the expected incidence rate, and the incidence rate of adenoid hypertrophy is 49.7%), a total of 120 mouth breathing children and adolescents were retrospectively examined and included in this study during the period from December 2018 to September 2019. |

Continued on next page

| Quantitative variables | 11 | Explain how quantitative variables were handled in the analyses. If applicable, describe which groupings were chosen and why | 3 | Independent samples t-tests were conducted to compare the difference between the measured index and the standard value, using SPSS software (version 22.0) for statistical analysis of measurements consistent with normality and homogeneity of variance. Differences were considered statistically significant when P<0.05. |
| --- | --- | --- | --- | --- |
| Statistical methods | 12 | (*a*) Describe all statistical methods, including those used to control for confounding |  |  |
|  |  | (*b*) Describe any methods used to examine subgroups and interactions |  |  |
|  |  | (*c*) Explain how missing data were addressed |  |  |
|  |  | (*d*) *Cohort study*—If applicable, explain how loss to follow-up was addressed  *Case-control study*—If applicable, explain how matching of cases and controls was addressed  *Cross-sectional study*—If applicable, describe analytical methods taking account of sampling strategy | 3 | Independent samples t-tests were conducted to compare the difference between the measured index and the standard value, using SPSS software (version 22.0) for statistical analysis of measurements consistent with normality and homogeneity of variance. Differences were considered statistically significant when P<0.05. |
|  |  | (*e*) Describe any sensitivity analyses |  |  |
| Results | | | | |
| Participants | 13* | (a) Report numbers of individuals at each stage of study—eg numbers potentially eligible, examined for eligibility, confirmed eligible, included in the study, completing follow-up, and analysed | 4 | According to the cervical vertebral maturation assessment, there were 45 CS1 cases, 33 CS2 cases, 21 CS3 cases, 9 CS4 cases, 12 CS5 cases, and 0 case of CS6. |
|  |  | (b) Give reasons for non-participation at each stage |  |  |
|  |  | (c) Consider use of a flow diagram |  |  |
| Descriptive data | 14* | (a) Give characteristics of study participants (eg demographic, clinical, social) and information on exposures and potential confounders | 3 | (1)7-15 age range; (2) Oral respiratory history≧2 years; (3) No family history of maxillofacial deformity; (4) No bad habits such as tongue spitting; (5) Lateral cephalograms of good quality; (6) Children and adolescents diagnosed as mouth breather. |
|  |  | (b) Indicate number of participants with missing data for each variable of interest |  |  |
|  |  | (c) *Cohort study*—Summarise follow-up time (eg, average and total amount) | 3 | from December 2018 to September 2019 |
| Outcome data | 15* | *Cohort study*—Report numbers of outcome events or summary measures over time |  |  |
|  |  | *Case-control study—*Report numbers in each exposure category, or summary measures of exposure |  |  |
|  |  | *Cross-sectional study—*Report numbers of outcome events or summary measures | *3* | *a total of 120 mouth breathing children and adolescents* |
| Main results | 16 | (*a*) Give unadjusted estimates and, if applicable, confounder-adjusted estimates and their precision (eg, 95% confidence interval). Make clear which confounders were adjusted for and why they were included |  |  |
|  |  | (*b*) Report category boundaries when continuous variables were categorized | 9 | Table 1 Indicator definitions and standard values were included |
|  |  | (*c*) If relevant, consider translating estimates of relative risk into absolute risk for a meaningful time period |  |  |

Continued on next page

| Other analyses | 17 | Report other analyses done—eg analyses of subgroups and interactions, and sensitivity analyses |  |  |
| --- | --- | --- | --- | --- |
| Discussion | | | | |
| Key results | 18 | Summarise key results with reference to study objectives | 4 | 3.1 Maxillofacial hard tissue measurements/3.2 Maxillofacial soft tissue measurements/ |
| Limitations | 19 | Discuss limitations of the study, taking into account sources of potential bias or imprecision. Discuss both direction and magnitude of any potential bias | 6 | It is significant to stress the caution that should be taken while interpreting the results presented in this study, for the lack of control group and its limitations as a cross-sectional study regarding growth analysis, which is lack of sensitivity to individual variability. And the results may be varied by region on account of genetics and nutrition. Thus, it is suggested that longitudinal studies are performed in different populations, investigating the changes in hard tissue, soft tissue, and airway measurements between cervical vertebral maturation stages of mouth breathing children and adolescents. |
| Interpretation | 20 | Give a cautious overall interpretation of results considering objectives, limitations, multiplicity of analyses, results from similar studies, and other relevant evidence | 6 | It is significant to stress the caution that should be taken while interpreting the results presented in this study, for the lack of control group and its limitations as a cross-sectional study regarding growth analysis, which is lack of sensitivity to individual variability. And the results may be varied by region on account of genetics and nutrition. Thus, it is suggested that longitudinal studies are performed in different populations, investigating the changes in hard tissue, soft tissue, and airway measurements between cervical vertebral maturation stages of mouth breathing children and adolescents. |
| Generalisability | 21 | Discuss the generalisability (external validity) of the study results | 6 | It is significant to stress the caution that should be taken while interpreting the results presented in this study, for the lack of control group and its limitations as a cross-sectional study regarding growth analysis, which is lack of sensitivity to individual variability. And the results may be varied by region on account of genetics and nutrition. Thus, it is suggested that longitudinal studies are performed in different populations, investigating the changes in hard tissue, soft tissue, and airway measurements between cervical vertebral maturation stages of mouth breathing children and adolescents. |
| Other information | |  | | |
| Funding | 22 | Give the source of funding and the role of the funders for the present study and, if applicable, for the original study on which the present article is based | 7 | This work was supported by Medical Research Key Project of Chongqing Science and Technology Commission and Health and Family Planning Commission Joint (Grant number 2018ZDXM020). |

*Give information separately for cases and controls in case-control studies and, if applicable, for exposed and unexposed groups in cohort and cross-sectional studies.

**Note:** An Explanation and Elaboration article discusses each checklist item and gives methodological background and published examples of transparent reporting. The STROBE checklist is best used in conjunction with this article (freely available on the Web sites of PLoS Medicine at http://www.plosmedicine.org/, Annals of Internal Medicine at http://www.annals.org/, and Epidemiology at http://www.epidem.com/). Information on the STROBE Initiative is available at www.strobe-statement.org.
